# Prognostic Value of Left Ventricular Diastolic Dysfunction in Patients Undergoing Cardiac Catheterization for Coronary Artery Disease

**DOI:** 10.1155/2012/243735

**Published:** 2012-04-12

**Authors:** Hidekatsu Fukuta, Nobuyuki Ohte, Kazuaki Wakami, Toshihiko Goto, Tomomitsu Tani, Genjiro Kimura

**Affiliations:** Department of Cardio-Renal Medicine and Hypertension, Nagoya City University Graduate School of Medical Sciences, 1 Kawasumi Mizuho-Cho Mizuho-Ku, Nagoya 467-8601, Japan

## Abstract

We hypothesized that left ventricular (LV) diastolic dysfunction assessed by cardiac catheterization may be associated with increased risk for cardiovascular events. To test the hypothesis, we assessed diastolic function by cardiac catheterization (relaxation time constant (Tau) and end-diastolic pressure (EDP)) as well as Doppler echocardiography (early diastolic mitral annular velocity (*e*′) and a ratio of early diastolic mitral inflow to annular velocities (*E*/*e*′)) in 222 consecutive patients undergoing cardiac catheterization for coronary artery disease (CAD). During a followup of 1364 ± 628 days, 5 cardiac deaths and 20 unscheduled cardiovascular hospitalizations were observed. Among LV diastolic function indices, Tau > 48 ms and *e*′ < 5.8 cm/s were each significantly associated with lower rate of survival free of cardiovascular hospitalization. Even after adjustment for potential confounders (traditional cardiovascular risk factors, the severity of CAD, and cardiovascular medications), the predictive value of Tau > 48 ms and *e*′ < 5.8 cm/s remained significant. No predictive value was observed in EDP, *E*/*e*′, or LV ejection fraction. In conclusion, LV diastolic dysfunction, particularly impaired LV relaxation assessed by both cardiac catheterization and Doppler echocardiography, is independently associated with increased risk for cardiac death or cardiovascular hospitalization in patients with known or suspected CAD.

## 1. Introduction

Left ventricular (LV) diastolic dysfunction refers to abnormalities in relaxation, filling, and distensibility [1]. Evidence of LV diastolic dysfunction can be determined by cardiac catheterization [1]. The presence of LV diastolic dysfunction can also be estimated by Doppler echocardiography [2, 3]. Although studies have shown that LV diastolic abnormalities assessed by Doppler echocardiography are predictive of adverse prognosis in various cardiac patients [4–10], the predictive value of invasively-determined diastolic dysfunction is unclear. In the present study, we hypothesized that invasively-determined diastolic dysfunction may be associated with increased risk for cardiovascular events. To test the hypothesis, we examined the association of LV diastolic dysfunction assessed by cardiac catheterization as well as Doppler echocardiography with subsequent cardiovascular events in patients undergoing cardiac catheterization for assessment of coronary artery disease (CAD).

## 2. Methods

### 2.1. Patients

We studied 222 consecutive Japanese patients who underwent cardiac catheterization for the evaluation of CAD between January 2004 and August 2006. All the patients had symptoms suggestive of angina and/or clinical signs of CAD (positive exercise electrocardiogram and/or abnormal myocardial perfusion scintigram). No patients with acute coronary syndrome, congestive heart failure, atrial fibrillation, primary valvular diseases, idiopathic dilated or hypertrophic cardiomyopathy, congenital heart disease, end-stage renal disease on maintenance hemodialysis, or malignant neoplasms were included. Medication status and a history of coronary revascularization were determined by review of medical records. Hypertension was defined as systolic blood pressure >140 mmHg and/or diastolic blood pressure >90 mmHg measured by indirect arm-cuff sphygmomanometry at rest or use of antihypertensive drugs. Diabetes was defined as a fasting blood glucose level >126 mg/dL or treatment with dietary modification, insulin, or oral hypoglycemic agents. Dyslipidemia was defined as low-density lipoprotein level ≥140 mg/dL, high-density lipoprotein level <40 mg/dL, and/or triglyceride level ≥150 mg/dL or treatment with antihyperlipidemic agents. Within a week before the index cardiac catheterization for this study, blood chemistry was obtained for assessment of clinical features. All the patients gave written informed consents to participate in the study, and this study was performed according to the regulations proposed by the Ethical Guidelines Committee of the Nagoya City University Graduate School of Medical Sciences.

### 2.2. Cardiac Catheterization

Before contrast material was injected into the LV or coronary artery, LV and aortic pressure waves were obtained with a catheter-tipped micromanometer (SPC-454D, Millar Instrument Company, Houston, TX) and recorded on a polygraph system (RMC-2000, Nihon Kohden, Inc., Tokyo, Japan) as previously reported [11]. From the recorded pressure waves, aortic systolic and diastolic pressures and LV end-diastolic pressure (EDP) were determined. A time constant of decrease in LV pressure (Tau), an index of early diastolic relaxation, was computed by applying a monoexponential fitting with zero asymptote to the LV pressure decay [12]. LV end-systolic and end-diastolic volumes were obtained from biplane left ventriculography by use of the method proposed by Chapman et al. [13] and were used for calculating ejection fraction (EF). The median values of measurements of 3 consecutive beats were used for statistical analyses.

### 2.3. Echocardiography

The day before the index cardiac catheterization, ultrasound examination was performed with the use of a commercially available echocardiographic machine (APLIO 80, Toshiba, Tokyo) with a 3-MHz transducer. Patients were examined at rest in the left lateral decubitus position. LV diastolic function was evaluated according to the published guideline [14]. Transmitral flow velocities during early diastole (*E*) and atrial contraction (*A*) at the mitral orifice were obtained with the use of pulsed Doppler echocardiography in the apical 4-chamber view. Deceleration time was measured as the time from the peak early filling velocity to termination of early filling. The peak early diastolic annular velocity (*e*′) was measured with the use of pulsed Doppler echocardiography at the septal and lateral mitral annular sites. The values of *e*′ measured at both sites were averaged. The median values of measurements of 3 consecutive beats were used for statistical analyses. LV mass was calculated from M-mode echocardiographic measurements [15] and LV mass was corrected for body surface area.

### 2.4. Followup

Followup was determined in October 2010 with the use of the medical record and/or at regular visit to determine the patients' vital status and any cardiovascular hospitalizations. The outcome of the present study was cardiac death (acute myocardial infarction, heart failure, and sudden cardiac death) or unscheduled admission for cardiovascular causes. Sudden cardiac death was defined as unexpected death within one hour after the onset of a new symptom, or unexpected, unobserved death.

### 2.5. Statistical Analysis

We used the SAS program package (SAS Institute, Cary, NC) for statistical analyses. Differences in quantitative and categorical data at baseline between groups were compared by the Student's *t*-test and the Fisher exact probability test. The association between continuous variables was determined by Pearson's correlation analysis. Survival curves of patients stratified by LV function indices were calculated by the Kaplan-Meier method and compared using the log-rank test. EDP of 16 mmHg, Tau of 48 ms, and EF of 50% were used as cut-off points, because these values are established thresholds for separating normal and abnormal levels [1]. In contrast, reported cut-off points of echocardiographic diastolic measures (*e*′ and *E*/*e*′) for the diagnosis of diastolic dysfunction or the prediction of adverse prognosis are variable depending on age [2] and population studied [4]. Furthermore, *e*′ recorded at lateral mitral annular site is usually higher than *e*′ at septal site [4]. Thus, in the present study, the best cut-off points of echocardiographic diastolic measures were explored to maximize the likelihood ratio in receiver-operating characteristic curves for the prediction of the outcome. Hazard ratio of clinical variables for the outcome was determined using the Cox proportional hazards regression analysis. To assess the independent predictive value of LV function indices, the multivariate Cox hazards regression analyses were performed including potential confounders based on the findings of previous studies and the results of univariate analyses. Multivariate model 1 included age, sex, hypertension, dyslipidemia, diabetes, and coronary revascularization after the index cardiac catheterization. Multivariate model 2 included previous history of myocardial infarction, the number of coronary arteries narrowed, EF, systolic aortic pressure, and heart rate. Multivariate model 3 included use of cardiovascular medications. Data are presented as the mean ± SD. Risk for death or cardiovascular hospitalization is presented as hazard ratio (HR) with 95% confidence interval (CI). *P* < 0.05 was considered significant.

## 3. Results

Based on the findings of the index coronary angiography, 50 percutaneous coronary interventions and 2 coronary artery bypass surgeries were performed. During a followup of 1364 ± 628  (median (range), 1444 (16–2433)) days, 5 cardiac deaths (4 heart failure and 1 sudden cardiac death) and 20 hospitalizations due to cardiovascular causes (15 ischemic myocardial events (14 unstable angina and 1 acute myocardial infarction), 3 heart failure, and 2 arrhythmia) were observed. Clinical, hemodynamic, and echocardiographic features of all patients and patient subgroups are shown in Table 1. Compared with patients who survived without cardiovascular hospitalization during the followup, those who died or were hospitalized due to cardiovascular causes were more likely to be older, to be prescribed statins, and to have higher levels of Tau and lower levels of *e*′ and were less likely to have hypertension. 

**Figure 1:**
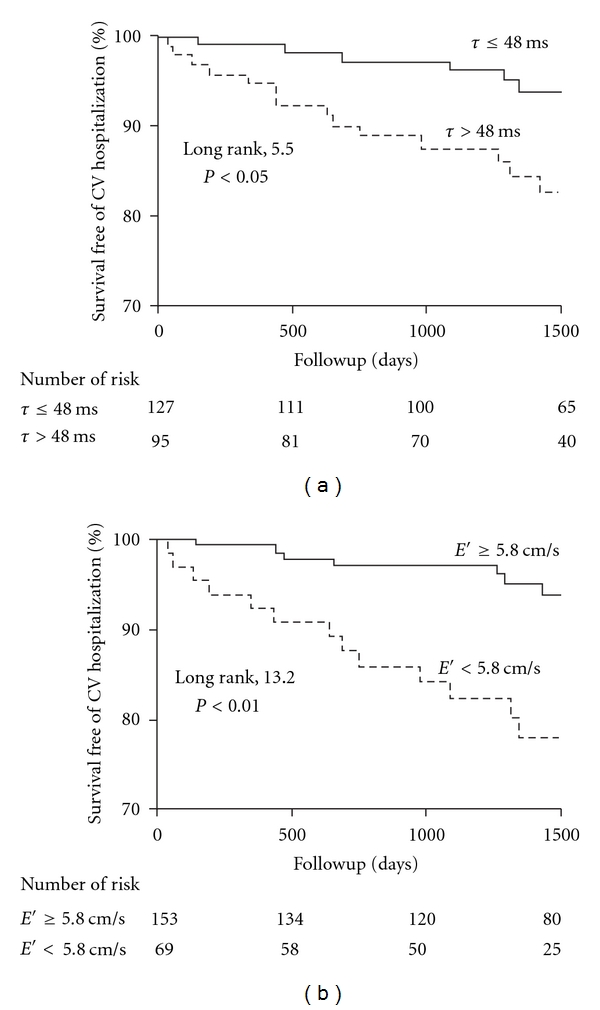
Kaplan-Meier survival curves of patients stratified by left ventricular relaxation time constant (Tau) and early diastolic mitral annular velocity (*e*′). CV indicates cardiovascular.

Based on receiver-operating characteristic analyses for the prediction of death or cardiovascular hospitalization, the best cut-off points for *e*′ and *E*/*e*′ were determined as 5.8 cm/s (sensitivity 56%, specificity 73%, likelihood ratio 2.06) and 13.2 (sensitivity 20%, specificity 88%, likelihood ratio 1.69), respectively. Among LV function indices, significant univariate predictors for death or cardiovascular hospitalization included Tau > 48 ms (HR [95% CI] = 2.57 [1.13–5.81], *P* < 0.05) and *e*′ < 5.8 cm/s (3.47 [1.57–7.70], *P* < 0.01). In contrast, no significant predictive value was observed in EDP > 16 mmHg (HR [95% CI] = 1.36 [0.61–3.0], *P* = 0.4), EF < 50% (0.83 [0.29–2.4], *P* = 0.7), or *E*/*e*′ > 13.2 (2.02 [0.76–5.42], *P* = 0.2). Survival curves of patients stratified by Tau and *e*′ are shown in Figure 1. Among other variables listed in Table 1, significant predictors for death or cardiovascular hospitalization included age (HR [95% CI] = 2.21 [1.31–3.71] per 1-SD increment, *P* < 0.01). 

Clinical features of patients stratified by Tau and *e*′ are shown in Table 2. Compared with patients with Tau ≤ 48 ms, those with Tau > 48 ms were more likely to have dyslipidemia and history of myocardial infarction, to be prescribed *β*-blockers, and to undergo coronary revascularization after the index cardiac catheterization. Compared with patients with *e*′ ≥ 5.8 cm/s, those with *e*′ < 5.8 cm/s were more likely to be older, to have diabetes and history of myocardial infarction, to be prescribed angiotensin converting enzyme inhibitors/angiotensin receptor blockers, and to undergo coronary revascularization after the index cardiac catheterization and were less likely to be prescribed calcium blockers.

Hemodynamic and echocardiographic features of patients stratified by Tau and *e*′ are also shown in Tables 2 and 3. Compared with patients with Tau ≤ 48 ms, those with Tau > 48 ms were more likely to have greater number of coronary arteries narrowed and higher levels of heart rate, EDP, and LV volumes and lower levels of systolic aortic pressure and EF. Compared with patients with *e*′ ≥ 5.8 cm/s, those with *e*′ < 5.8 cm/s were more likely to have greater number of coronary arteries narrowed and higher levels of Tau, EDP, and LV volumes and lower levels of EF. 

Patients who were diagnosed to have abnormal LV relaxation by both cardiac catheterization and Doppler echocardiography (Tau > 48 ms and *e*′ < 5.8 cm/s (*n* = 41)) were more likely to be older, to have history of myocardial infarction, to be higher in serum creatinine levels, LV volume, and LV mass index, and to be lower in EF, compared with those who were diagnosed to have abnormal LV relaxation by only one modality (*n* = 82).

After adjustment for potential confounders (traditional cardiovascular risk factors, the severity of CAD, and cardiovascular medications, coronary revascularization after the index cardiac catheterization), the predictive value of Tau > 48 ms continued to be significant (Table 4). Similar adjustment revealed the independent predictive value of *e*′ < 5.8 cm/s (Table 4).

Modest but significant correlations were observed between LV diastolic function indices determined by cardiac catheterization and those by Doppler echocardiography; Tau correlated with *e*′ (*r* = −0.26, *P* < 0.001) and *E*/*e*′ (*r* = 0.26, *P* < 0.001) and EDP correlated with *E*/*e*′ (*r* = 0.23, *P* < 0.001) but not with *e*′ (*r* = −0.08, *P* = 0.3).

## 4. Discussion

In the present study, we found that Tau > 48 ms and *e*′ < 5.8 cm/s were each associated with an increased risk for cardiac death or subsequent cardiovascular hospitalization in patients undergoing cardiac catheterization for CAD. In contrast, no prognostic value was observed in EDP, *E*/*e*′, or EF.

Although studies have reported that LV diastolic abnormalities assessed by Doppler echocardiography are predictive of cardiac mortality and morbidity in patients with myocardial infarction [5], those with heart failure with preserved EF [7], those with reduced EF [8], and hypertensive subjects [6, 9], only a few studies have examined the predictive value of invasively-determined diastolic dysfunction. Specifically, Liang et al. examined the prognostic value of EDP as well as Doppler echocardiographic diastolic measures in patients undergoing cardiac catheterization for CAD [16]. They found that EDP > 20 mmHg and *E*/*e*′ ≥ 15 were each predictive of future heart failure events. In contrast, in our study, no prognostic value was observed in EDP or *E*/*e*′. Compared with a cohort of Liang et al., our patients had lower EDP (18.2 ± 7.3 versus 14.4 ± 5.4 mmHg) and *E*/*e*′ (12.5 ± 5.6 versus 9.6 ± 3.3) levels, due probably to that patients with congestive heart failure were not included in our study. Thus, EDP or *E*/*e*′ may not be predictive of adverse prognosis in a cohort of patients without congestive heart failure in which the majority have normal or slightly elevated EDP and *E*/*e*′.

The strength of the present study is that LV hemodynamic variables were obtained with the use of a micromanometer catheter but not a fluid-filled catheter. Although a fluid-filled catheter accurately measures late diastolic LV pressures, it cannot precisely determine rapidly changing pressures as occur during LV isovolumetric relaxation [17]. Use of a micromanometer catheter enabled us to determine Tau, an index of LV relaxation. Although LV relaxation can be estimated from *e*′ on Doppler echocardiography, *e*′ quantitates the peak velocity of early diastolic longitudinal motion of the mitral annulus [18] and measurement of *e*′ only provides best-available noninvasive assessment of LV relaxation. Our study is significant in showing for the first time the prognostic value of abnormal LV relaxation determined by cardiac catheterization in patients with known or suspected CAD.

Although the present study does not provide direct mechanisms underlying the association between abnormal LV relaxation and adverse prognosis in patients with known or suspected CAD, there are possible explanations. The most common cause of cardiac death or cardiovascular hospitalization in our study was ischemic myocardial events. It is well-established that the presence of myocardial ischemia impairs LV relaxation [19]. In fact, we observed that patients with impaired LV relaxation had more severe CAD (Table 2). Thus, the prognostic association of impaired LV relaxation may be mediated at least in part by CAD severity. We observed, however, that after adjustment for the index coronary angiographic findings, the predictive value of impaired LV relaxation remained significant (Table 4, Model 2). Thus, a part of the prognostic association of impaired LV relaxation may be due to inducible myocardial ischemia regardless of the stenoses of epicardial coronary arteries. Consistent with this explanation, studies have shown that impaired LV relaxation diminishes early diastolic coronary flow [20, 21] and may thereby further exaggerate myocardial ischemia particularly when accompanied by tachycardia (such as tachyarrhythmia and during exertion).

Another possible explanation for our observed association of impaired LV relaxation with adverse prognosis is that LV diastolic abnormalities may coexist with other disorders that may adversely impact on prognosis. Specifically, Lee et al. reported that altered LV diastolic function assessed by Doppler echocardiography was independently associated with endothelial dysfunction and hemostatic abnormalities [22], both of which have been reported to be predictive of the development of atherosclerotic diseases and ischemic myocardial events in patients with CAD [23, 24].

There are several limitations in the present study. First, LV mass was available in the limited number of the patients (68%), which could have influenced our results [25]. Furthermore, estimation of LV mass by M-mode echocardiography in the presence of altered LV geometry such as myocardial infarction may be inappropriate [15]. Second, we did not measure left atrial volume index, a powerful predictor for left atrial pressure and clinical outcomes [14, 26]. Third, the time difference between cardiac catheterization and Doppler echocardiographic examination is not small (about one day). This may explain our observed modest correlations of diastolic function indices obtained from cardiac catheterization with those from Doppler echocardiography and may also possibly explain the lack of prognostic value of *E*/*e*′. Forth, our study patients were composed of stable known or suspected CAD patients without congestive heart failure who were referred for cardiac catheterization for assessment of CAD. Our findings cannot be extended to other populations. Finally, our study is not large enough to permit us to evaluate the association of LV diastolic dysfunction with specific causes of death or hospitalization.

Our study suggests that measurement of *e*′ by Doppler echocardiography may provide useful information for risk stratification in known or suspected CAD patients without congestive heart failure. The cut-off point of *e*′ used in the present study, however, is higher than that previously reported. Specifically, Wang et al. reported that *e*′ < 3 cm/s was a powerful predictor for cardiac death in patients with reduced (<50%) EF [8]. Similarly, the same group reported the predictive value of *e*′ < 3.5 cm/s for cardiac death in hypertensive subjects with LV hypertrophy [9]. These results suggest that the best cut-off point of *e*′ for the prediction of adverse prognosis may be variable depending on population studied. Further studies are necessary for determining the optimal cut-off points of *e*′ for identifying high-risk patients in various populations.

Our findings suggest that patients with impaired LV relaxation may need more aggressive treatment of CAD. A recent study has reported that altered LV relaxation assessed by Doppler echocardiography is improved after coronary revascularization in patients with ischemic cardiomyopathy [27]. The prognostic impact of improved LV relaxation after coronary revascularization in CAD patients merits future research.

## Figures and Tables

**Table 1:** Clinical, hemodynamic, and echocardiographic features of all patients and patient subgroups.

Variables	All patients (*n* = 222)	Outcomes*, no (*n* = 197)	Outcomes, yes (*n* = 25)	*P* value^†^
Age, year	67 ± 8	66 ± 8	71 ± 7	<0.01
Men	78%	79%	76%	NS
Body mass index, kg/m^2^	24.1 ± 3.2	24.1 ± 3.3	24.3 ± 2.8	NS
Hypertension	49%	52%	29%	<0.05
Diabetes	40%	39%	50%	NS
Dyslipidemia	86%	84%	96%	NS
Previous myocardial infarction	60%	59%	68%	NS
Previous coronary revascularization	46%	45%	56%	NS
Coronary revascularization after the index cardiac catheterization	23%	22%	32%	NS
Serum creatinine, mg/dL	0.84 ± 0.20	0.84 ± 0.20	0.86 ± 0.20	NS
Medications				
Angiotensin converting enzyme inhibitors/angiotensin receptor blockers	43%	45%	32%	NS
*β*-blocker	41%	42%	38%	NS
Calcium blocker	32%	34%	20%	NS
Statin	58%	56%	79%	<0.05
Antiplatelet agents	81%	80%	92%	NS
Number of coronary arteries narrowed^‡^				NS
0	25%	26%	12%	
1	26%	24%	40%	
>1	49%	49%	48%	
Heart rate, beat/min	68 ± 12	68 ± 12	65 ± 12	NS
Systolic aortic pressure, mmHg	139 ± 25	139 ± 25	140 ± 21	NS
Diastolic aortic pressure, mmHg	68 ± 11	69 ± 11	65 ± 11	NS
Left ventricular end-diastolic pressure, mmHg	14.4 ± 5.4	14.3 ± 5.2	15.0 ± 5.9	NS
Relaxation time constant (Tau), ms	46.4 ± 9.1	46.0 ± 9.1	49.5 ± 8.4	0.06
Left ventricular end-diastolic volume index, mL/m^2^	85.2 ± 21.0	84.5 ± 21.4	90.1 ± 22.7	NS
Left ventricular end-systolic volume index, mL/m^2^	34.0 ± 19.2	33.5 ± 19.4	37.6 ± 18.0	NS
Ejection fraction	62 ± 13%	62 ± 13%	60 ± 11%	NS
Echocardiographic indices				
E/A	0.84 ± 0.41	0.84 ± 0.42	0.82 ± 0.34	NS
Deceleration time, ms	211 ± 50	213 ± 51	200 ± 45	NS
*e*′ cm/s	7.0 ± 2.0	7.1 ± 2.1	6.4 ± 1.7	0.06
*E*/*e*′	9.6 ± 3.3	9.5 ± 3.4	10.0 ± 2.8	NS
Left ventricular mass index, g/m^2¶^	108.6 ± 27.6	107.2 ± 26.9	121.6 ± 30.9	0.05

Data are expressed as the mean ± standard deviation or frequency.

*Outcomes were defined as cardiac death (acute myocardial infarction, heart failure, and sudden cardiac death) or unscheduled admission for cardiovascular causes.

^†^Outcomes yes versus no.

^‡^Narrowed coronary artery was defined as major epicardial artery with ≥75% stenosis on angiogram.

^¶^Left ventricular mass was available in 152 patients.

**Table 2:** Clinical and hemodynamic features of patient subgroups stratified by left ventricular relaxation time constant (Tau) and peak early diastolic mitral annular velocity (*e*′).

	Tau			*e*′		
	≤48 ms	>48 ms	*P* Value	≥5.8 cm/s	<5.8 cm/s	*P* value
	(*n* = 127)	(*n* = 95)		(*n* = 153)	(*n* = 69)	
Age, year	67 ± 8	67 ± 9	NS	65 ± 8	70 ± 6	<0.001
Men	76%	82%	NS	80%	75%	NS
Body mass index, kg/m^2^	23.8 ± 3.1	24.5 ± 3.3	NS	23.9 ± 3.3	24.6 ± 3.0	NS
Hypertension	47%	53%	NS	52%	49%	NS
Diabetes	40%	41%	NS	35%	53%	<0.05
Dyslipidemia	80%	94%	<0.01	83%	91%	NS
Previous myocardial infarction	48%	76%	<0.001	53%	75%	<0.01
Previous coronary revascularization	42%	53%	NS	44%	52%	NS
Coronary revascularization after the index cardiac catheterization	18%	31%	<0.05	19%	33%	<0.05
Serum creatinine, mg/dL	0.83 ± 0.19	0.85 ± 0.21	NS	0.83 ± 0.19	0.87 ± 0.20	NS
Medications						
Angiotensin converting enzyme inhibitors/angiotensin receptor blockers	39%	48%	NS	38%	55%	<0.05
*β*-blocker	30%	56%	<0.001	41%	41%	NS
Calcium blocker	35%	29%	NS	37%	23%	<0.05
Statins	52%	66%	NS	58%	60%	NS
Antiplatelet agents	77%	87%	<0.05	81%	82%	NS
Number of coronary arteries narrowed			<0.01			<0.01
0	32%	15%		31%	12%	
1	26%	26%		26%	26%	
>1	42%	59%		43%	62%	
Heart rate, beat/min	71 ± 12	64 ± 10	<0.001	68 ± 12	69 ± 11	NS
Systolic aortic pressure, mmHg	142 ± 27	134 ± 21	<0.05	137 ± 24	143 ± 27	0.07
Diastolic aortic pressure, mmHg	69 ± 11	67 ± 11	NS	68 ± 10	68 ± 12	NS
Left ventricular end-diastolic pressure, mmHg	12.3 ± 3.8	17.2 ± 5.7	<0.001	14.2 ± 5.2	14.8 ± 5.4	NS
Relaxation time constant (Tau), ms	40.2 ± 5.1	54.6 ± 6.1	<0.001	45.2 ± 9.3	48.9 ± 8.1	<0.01
Left ventricular end-diastolic volume index, mL/m^2^	77.0 ± 16.3	96.1 ± 23.1	<0.001	80.3 ± 17.4	95.9 ± 25.8	<0.001
Left ventricular end-systolic volume index, mL/m^2^	26.6 ± 12.2	43.9 ± 22.3	<0.001	29.2 ± 15.0	44.5 ± 23.1	<0.001
Ejection fraction, %	66 ± 10	56 ± 14	<0.001	65 ± 12	56 ± 14	<0.001

**Table 3:** Echocardiographic feature of patient subgroups stratified by left ventricular relaxation time constant (Tau) and peak early diastolic mitral annular velocity (*e*′).

		Tau			*e*′	
	≤48 ms	>48 ms	*P* value	≥5.8 cm/s	<5.8 cm/s	*P* value
	(*n* = 127)	(*n* = 95)		(*n* = 153)	(*n* = 69)	
Echocardiographic indices						
E/A	0.79 ± 0.23	0.90 ± 0.55	0.05	0.87 ± 0.33	0.77 ± 0.54	NS
Deceleration time, ms	218 ± 48	201 ± 52	NS	210 ± 51	214 ± 49	NS
*e*′ cm/s	7.5 ± 2.0	6.4 ± 1.9	<0.001	8.0 ± 1.6	4.8 ± 0.8	<0.001
*E*/*e*′	8.8 ± 2.8	10.6 ± 3.7	<0.001	8.3 ± 2.3	12.4 ± 3.6	<0.001
Left ventricular mass index, g/m^2^	99.8 ± 24.5	120.7 ± 26.9	<0.001	101.5 ± 25.4	122.9 ± 26.5	<0.001

**Table 4:** Unadjusted and adjusted hazard ratios of left ventricular relaxation time constant (Tau) > 48 ms and peak early diastolic mitral annular velocity (*e*′) < 5.8 cm/s by Cox proportional hazards regression analysis.

	Unadjusted		Adjusted	
		Model 1*	Model 2^†^	Model 3^‡^
Tau > 48 ms	2.57 (1.13–5.81)^¶^	2.45 (1.04–5.82)^¶^	3.12 (1.16–8.37)^¶^	3.23 (1.31–7.97)^¶^
*e*′ < 5.8 cm/s	3.47 (1.57–7.70)^#^	2.38 (1.03–5.52)^¶^	4.09 (1.75–9.57)^#^	3.81 (1.65–8.81)^#^

Data are represented as hazard ratio (95% CI).

*Adjusted for age, sex, hypertension, dyslipidemia, diabetes, and coronary revascularization after the index cardiac catheterization.

^†^Adjusted for previous history of myocardial infarction, the number of coronary arteries narrowed, ejection fraction, systolic aortic pressure, and heart rate.

^‡^Adjusted for use of *β*-blocker, angiotensin-converting enzyme inhibitors or angiotensin receptor blockers, calcium blocker, statin, and antiplatelet agents.

^¶^ *P* < 0.05.

^#^ *P* < 0.01.
